# The importance of mentoring during educational supervision

**DOI:** 10.1007/s40037-016-0291-3

**Published:** 2016-08-04

**Authors:** Vimmi Passi

**Affiliations:** Buckinghamshire, United Kingdom

Educational supervision is regular supervision taking place in the context of a recognized training programme in order to determine learning needs and review progress. Patel’s article [1] effectively evaluates postgraduate educational supervision in one UK trust. This was a mixed methods study in which educational supervisors and trainees working within a large UK trust were surveyed online about their experiences of educational supervision. In addition, observations of supervision sessions with a small group of supervisor and trainee pairs followed up by semi-structured interviews were conducted. The findings suggested methods to improve educational supervision and a framework was developed [1]. The framework illustrates the need to improve student support during educational supervision [1].

This commentary explores the importance of mentoring during educational supervision. A mentorship is a fortuitous relationship that fosters the development of the adult learner [2]. Educational supervision offers the unique opportunity to be an effective mentor to the student. The mentoring can be informal or more formal within the scheduled supervision meetings. Mentoring should be recognized as an important teaching activity [3]. During the initial educational supervision meetings, it is important to establish an effective relationship with the leaner and provide feedback on their current progress before offering specific guidance. Effective feedback is non-judgmental, timely and accompanied by reflection to help the learner improve [4]. The educational supervisor can then provide specific guidance to enhance academic, professional and personal development.

Firstly, with regard to academic development, the educational supervisor can discuss learning styles, progress with training and time management. It is valuable to discuss the learner’s personal development plan to check that this is specific, relevant and realistic. The personal development plan should include any relevant courses to attend and preparation for postgraduate exams. Educational supervision also offers the opportunity for reflection on career plans as students may be considering specific speciality choices. Career choices in medicine can be challenging for the trainee and supervisors can offer expertise regarding the different specialities, preparing the curriculum vitae, application processes and requirements.

Secondly, with regard to professional development, it is important that the supervisor ensures the learner demonstrates high standards of medical professionalism. There are numerous definitions of medical professionalism provided by major medical organizations. In the UK, the Royal College of Physicians of London’s Working Party on Medical Professionalism has defined medical professionalism succinctly as ‘a set of values, behaviours and relationships that underpin the trust the public has in doctors’ with doctors being committed to integrity, compassion, altruism, continuous improvement, excellence and teamwork’ [5]. The educational supervisor has the unique opportunity to read the workplace-based assessments of the trainee and can review the multisource feedback report and patient satisfaction reports, both of which offer information regarding the professional attributes of the trainee.

Thirdly, with regard to a trainee’s personal development, the educational supervisor can offer pastoral support. This is particularly important for students who may be experiencing some difficulties during their training and in these circumstances the supervisor should demonstrate compassion, empathy and a non-judgemental, confidential approach. In addition, a new emerging theme in medical education is the importance of developing resilience [6]. Resilience has been described as an emotional competence and can be considered as behaviour to be acquired during training [7]. Educational supervisors can offer suggestions to develop resilience, avoid burnout and maintain a healthy work-life balance during training.

In summary, the educational supervision offers an important opportunity for effective mentoring. Educational supervisors should ensure high standards of mentoring and student support throughout the curriculum. The trainees should make best use of this support to ask questions, to discuss any concerns, to seek professional and career advice.

Observe, record, tabulate, and communicate. Use your five senses. Learn to see, learn to hear, learn to feel, learn to smell and know that by practice alone you can become an expert. (William Osler 1849–1919).
